# Study the Effect of an Innovative Educational Program Promoting Healthy Food Habits on Eating Disorders, Mediterranean Diet Adherence and Body Composition in University Students

**DOI:** 10.3390/healthcare11070965

**Published:** 2023-03-28

**Authors:** Alejandro Martínez-Rodríguez, Lorena Vidal-Martínez, María Martínez-Olcina, Laura Miralles-Amorós, Juan Antonio Sánchez-Sáez, Domingo Jesús Ramos-Campo, Javier Sánchez-Sánchez, Natalia Martínez-Amorós, Kamela Cheikh-Moussa, Nuria Asencio-Mas, Luis Andreu-Caravaca, Jacobo Ángel Rubio-Arias

**Affiliations:** 1 Department of Analytical Chemistry, Nutrition and Food Sciences, University of Alicante, 03690 Alicante, Spain; 2 Faculty of Sport, Catholic University of Murcia, 30107 Murcia, Spain; 3 Department of Health and Human Performance, Faculty of Physical Activity and Sport Science, Polytechnic University of Madrid, 28040 Madrid, Spain; 4 School of Sport Sciences, Universidad Europea de Madrid, 28670 Madrid, Spain; 5 Health Research Center, Department of Education, Faculty of Educational Sciences, University of Almería, 04120 Almería, Spain

**Keywords:** nutrition degree students, eating disorders, body composition, fat mass, adherence to the Mediterranean diet

## Abstract

The university stage is a good time to promote healthy eating strategies. The sociological and cultural changes experienced by students lead them to skip meals, increase their intake of fast food and energy-dense foods, decreasing adherence to the Mediterranean diet (MD). Professionals related to food such as nutritionists and dietitians are also considered a population at risk of developing eating disorders due to the extensive knowledge they possess, which can be used for both good and bad practice. The objective was to analyze the impact of a 4-month educational program promoting the Mediterranean diet on risky eating behaviors, adherence to the Mediterranean diet, and body composition in nutrition and dietetics students, studying the differences according to the group investigated (control group and educated group). The context of the research was 196 students (49 males and 147 females) from two consecutive years. The control group did not receive any type of intervention, while with the control group (educated) an educational program was carried out. Results showed that women with greater control over energy intake, carbohydrates, and sugar had a lower percentage of fat mass, while in men, the relationship was established with weight. Regarding adherence to the Mediterranean diet, at post, it is adequate in both men (8.25 ± 2.87) and women (7.90 ± 2.89), with no significant differences between the intervention groups.

## 1. Introduction

The college years fall during a crucial developmental phase known as emerging adulthood [1]. During this age, previous studies have shown that the incidence of eating disorders peaks in late adolescence. It is estimated that 2–4% of young adults suffer from eating disorders (EDs) [2]. Additionally, EDs are associated with several physiological alterations, comorbidities, and an increased risk of mortality [3]. The mortality rate of people with ED is the highest among all psychiatric illnesses. It is estimated to be twelve times higher than that of other psychiatric illnesses [4]. ED detection rates in different populations range from 4.5% to 6.2% in China [5] to 24.8% in France [6], with Spain having one of the highest rates (20.8%) [7].

This increase in EDs during adolescence could be because during this period of life, the university population is subject to several sociological and cultural changes. In this regard, many students move away from their families, leave home, begin college education, become responsible for their own eating habits, organizing their time, buying food, preparing their meals, and organizing meal schedules [8,9]. All of these factors can lead to the regular skipping of meals, a preference for fast food, alcohol consumption, smoking, and ultimately, to the development changes in weight and body composition [8]. This period of life also coincides with the peak of onset of many mental and EDs [10], which could therefore be a relevant target stage for intervention to improve their lifestyle habits and to build healthy eating habits.

Among the population at risk are students in food-related careers. Freitas et al. [11] reported that food-related professionals such as nutritionists and dietitians should be considered at risk of developing eating disorders. In fact, in a recent systematic review with a meta-analysis [12], they observed that the rate of nutrition and dietetics students with a positive eating attitude test (EAT-26; cutoff ≥ 21 points) was significantly higher than all other majors. Considering the impact of this issue on the performance of these health professionals, it is relevant to study eating disorders in this population. Several research and systematic reviews have shown that nutrition students obtain higher scores in screening tests for EDs [9,13].

Even considering that nutrition and dietetics is an area prone to a higher prevalence or predisposition to eating disorders, the etiology of this relationship is not yet fully explained [14]. It is unknown whether the increased prevalence of eating disorders is due to increased attendance at nutrition or dietetics courses by individuals motivated by their experiences in nutrition and weight control, or contact with these topics during the course, and their belief that a good appearance is important for future career success is the source of this problem [15,16,17].

Some studies have analyzed the relationship between EDs and body composition in nutrition and dietetics students, however, the analysis was limited only to body mass index (BMI) or waist/hip ratios, without further consideration of other variables such as percentage (%) of fat mass (FM) or muscle mass (MM) [8,18,19,20].

These circumstances condition the teachers’ need to investigate the current situation of eating habits, body composition, and the risk of suffering from ED in this community. It is vital to transfer to nutrition and dietetics students the importance of good dietary and nutritional education, introducing this knowledge in an innovative way. It will be relevant to integrate within the students an assessment of themselves, making them aware of different aspects and behaviors that can cause ED. This will help the students, who, integrating this problem, will be able to identify and treat it better, preventing the development of eating disorders when in clinical practice. This knowledge can be transformed in an adequate nutritional status, promoting healthy habits in the population with whom they work.

The Mediterranean diet (MD) could be a key intervention among nutritional strategies as this eating style is a protective factor against obesity or non-communicable diseases such as type II diabetes mellitus [21], stroke, and cardiovascular problems [22]. The MD is characterized by being a balanced diet that provides sufficient energy in the right proportions through the high consumption of vegetables, legumes, fruits, nuts, cereals, and olive oil, a moderate consumption of fish, eggs, and dairy products, preferably yogurt or cheese, and a lower intake of meat and less consumption of animal fats [23].

The Mediterranean diet is also characterized by using different herbs and plants such as basil, bay leaves, mint, rosemary, and sage to enhance food flavor and taste perception. These have a high capacity to impart distinctive aromas, which can modulate the perception of salty taste by providing proteins, fiber, volatile components (essential oils), vitamins, minerals, phytochemicals, and contribute significantly to the promotion of human health due to their different beneficial properties (antioxidant activity, anticancer activity, and prevention of cardiovascular diseases) and neurodegenerative diseases [24].

The university stage is a good time to promote healthy eating strategies, since the increase in the consumption of energy-dense and unstable diets shows a low adherence to the MD among students [8]. A study of 597 university students between 17 and 20 years old, in southern Spain showed that 21.9% needed to improve their diet [25]. In this context, the objectives of the research were (a) to analyze the impact of an educational program of 4 months promoting the Mediterranean diet on body composition, the risk of suffering eating disorders, and adherence to a healthy diet in male and female students enrolled in the Fundamentals of Human Nutrition and Dietetics course at the University of Alicante, in the province of Alicante, Spain; and (b) to analyze the relationship between the variables of risk to develop an eating disorder, adherence to Mediterranean diet, and body composition.

## 2. Materials and Methods

### 2.1. Study Design

The research was conducted during the first semester of the academic years 2021/22 and 2022/23 at the University of Alicante. An intra- and inter-subject quasi-experimental design with a pre- and post-test was conducted to identify the effects of a 4-month educational program promoting healthy eating styles on risky eating behaviors, adherence to the Mediterranean diet, and body composition. For this purpose, a group of students enrolled in the nutrition and dietetics degree was evaluated during an academic year in which they did not receive any educational program and the following year in which they received a specific nutritional educational program.

A total of 196 first-year nutrition and dietetics students were recruited through non-probability sampling: 49 males (20.8 ± 6.72 years) and 147 females (19.6 ± 2.90 years). The 2021/22 group participated as a control group (no educational intervention, CG), while the 2022/23 group received an educational intervention (EIG). The nutrition degree students who had not attended the practical classes were excluded, therefore, they could not partake in the body composition measurements. The study was conducted in accordance with the Declaration of Helsinki. All procedures were previously approved by the Ethics Committee of the University of Alicante (UA-2021-03-11).

### 2.2. Experimental Procedures

The risk of developing an eating disorder (EAT-26 questionnaire), composition measured with Biodyxpert^®^, and adherence to the Mediterranean diet (PREDIMED questionnaire) were analyzed at the baseline and at 4 months in both academic years. Questionnaires were completed by the students as an online survey given their convenient and anonymous format. A qualified nutritionist analyzed both questionnaires and calculated the scores. The analysis of body composition was performed in class, in the practical work of the Fundamentals of Nutrition and Dietetics in the first year of the degree.

In the CG, in the last practical, they were given a complementary lecture to what they studied in class to increase their awareness of the importance of a good dietetic-nutritional education. In this session, the students were shown the results obtained after performing the tests by themselves (both on the first day of class and after 6 months), making them aware and able to quickly and effectively identify the risk behaviors that can result in an eating disorder. In the EIG, an educational program was designed parallel to the classes they attended, related to diet, lifestyle, and risks of eating disorders. The program included three topics (Table 1); each topic began by talking about “myths” established today and ended by giving scientific answers, based on previous research [26,27]. Each topic lasted approximately 45–60 min and was provided by three researchers. The three educational workshops were developed in the first month.

### 2.3. Materials

The EAT 26 questionnaire was used to screen the potential risk of developing an eating disorder [29]. It consists of 26 items divided into three scales: dieting (food restriction and obsession to lose weight), bulimia (use of binge eating/vomiting induced behaviors and thoughts about food), and preoccupation with food and oral control (self-control of food intake and environmental pressure to lose weight) (Cronbach’s alpha > 0.78) [29]. In the CG, these results were presented to the nutrition and dietetics students in terms of contributing to their auto-perception about the different behaviors related to eating disorders. In the EIG, the results of the baseline measurement were shown in educational workshop 3. This allowed them to use that experience as an innovative teaching action to develop knowledge integration about dietary habits to prevent eating disorders in future patients.

The Biodyxpert^®^ (La Ciotat, France) was used to measure body composition. This impedance meter allows for an accurate analysis of body composition with professional hand/foot, multi-frequency, and multi-algorithm technology. It consists of passing a low intensity alternating electric current through the body that measures the opposition of the tissues to the passage of this current. The value of the impedances, phase angle, resistances and reactance collected by the measuring device allow, thanks to algorithms, for the determination of body compartments crossed by the current and provides a detailed body composition. The current used was of very low intensity and completely painless.

Adherence to MD was assessed using the 14-item questionnaire previously validated for the evaluation of prevention with the Mediterranean diet (PREDIMED) [30]. A score of 1 and 0 was assigned for each item; the total PREDIMED score was calculated as follows: score 0–5, low adherence; score 6–9, medium adherence; score > 10, higher adherence [30].

### 2.4. Statistical Analysis

Descriptive statistics including the means and standard deviations (SD) were used. The Shapiro–Wilk test was performed to evaluate the normality of the descriptive statistics. To calculate the effect of the educational program, analysis of variance (ANOVA) was used. Post hoc tests (Bonferroni) were performed when significant interaction (group × time) effects were observed. Eta squared partial (η^2^p) for variance analysis calculated the effect size (η^2^p ≥ 0.01 indicates a small effect, ≥0.059 a medium effect, and ≥0.138 a high effect). Finally, linear relationships between pairs of continuous variables were analyzed using Pearson’s correlation coefficient. In addition, the following cut-off points suggested by Cohen (1988) for correlations were established: 0.10–0.29 small association; 0.30–0.49 moderate correlation, and ≥0.50 strong or large correlation [31]. A level of *p*  <  0.05 established statistical significance. All analyses were performed using the Mac version of the JAMOVI statistical program (version 1.6.15, The JAMOVI Project, Sydney, Australia).

## 3. Results

**Figure 1:**
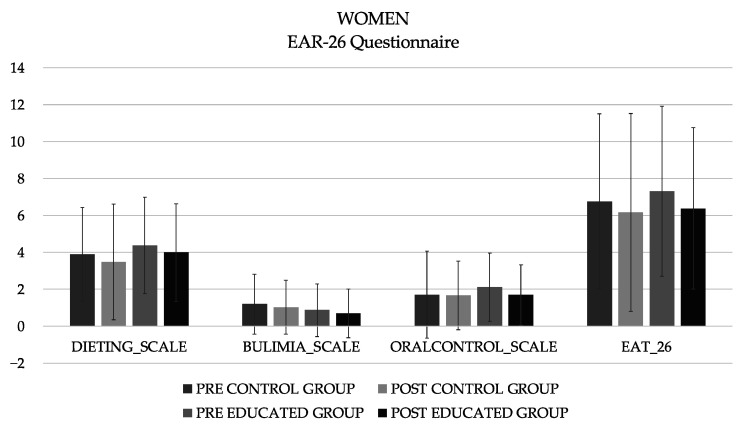
Descriptive values (mean) of the different subscales and total of the EAT-26 questionnaire in women.

**Figure 2:**
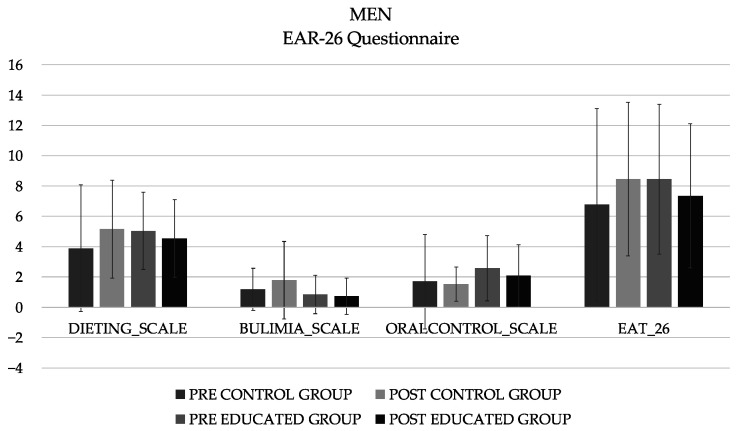
Descriptive values (mean) of the different subscales and total of the EAT-26 questionnaire in men.

A total of 196 first-year nutrition and dietetics students participated, and the CG included 23 men (20.3 ± 7.20 years) and 62 women (19.6 ± 3.51 years; 163 ± 5.86 cm height), and the EIG included 26 men (21.4 ± 6.35 years) and 65 women (19.6 ± 1.88 years; 162 ± 6.15 cm height). The analysis of the differences in the different subscales of the EAT-26, and total sum showed no significant differences in either the women (Figure 1) or men (Figure 2).

For women (Table 2), the main effects were observed for all body composition variables. The Bonferroni test showed a decrease in the EIG group for weight (*p* = 0.001), BMI (*p* < 0.001), fat mass at constant hydration (FMCH) (*p* = 0.001), and crude fat mas (CFM) (*p* < 0.001). However, there has also been an increase in skeletal muscle mass (SMM) (*p* < 0.001), appendicular skeletal muscle mass (ASMM) (*p* < 0.001), hydration without grass (HWG) (*p* = 0.001), and total water (*p* = 0.002). In addition, it showed differences between CG and EIG in the post moment for CFM (*p* < 0.001), SMM (*p* < 0.001), ASMM (*p* < 0.001), and water (*p* = 0.003).

In men (Table 3), there were statistically significant differences in the variables SMM and HWG (*p* < 0.005). After performing the post hoc analysis, a trend was observed in the CFM variable (*p* = 0.052) between the pre and post of the educated group. Regarding SMM, in the educated group, the post results were significantly higher than the pre (*p* = 0.034). However, there are differences both at the beginning (*p* < 0.001) and at the end (*p* < 0.001) between both groups, with the control group having higher SMM and ASMM values.

As for the PREDIMED (Table 4), significant differences were observed in men and women, both in the control group (*p* < 0.001) and in the educated group (*p* < 0.001), with the pre values significantly lower than the post in both cases.

The correlations of the different variables included in the research are presented in Table 5 (women) and Table 6 (men). In the case of the women, a small negative correlation was observed between the oral control subscale and the weight variable (*p* = 0.002) and between the dieting subscale (*p* = 0.030) and total score of the EAT-26 with FMCH (*p* = 0.049).

In men, moderate negative correlations were observed between the different body composition variables and the PREDIMED results: weight (*p* = 0.001), BMI (*p* < 0.001), FMCH (*p* = 0.014), and CFM (*p* = 0.004). Regarding the EAT-26 questionnaire, moderate negative correlations were found between the dieting scale with respect to weight (*p* = 0.033) and BMI (*p* = 0.005) and between the total score and BMI (*p* = 0.011).

## 4. Discussion

The main objective of this research was to test the effects of an educational intervention based on nutrition, healthy habits, and eating disorders on the risk of eating disorders (EAT-26), body composition, and adherence to the Mediterranean diet (PREDIMED), in addition to establishing correlations between these variables.

From the total number of nutrition and dietetics students who participated, one student in each group was at high risk for eating disorders (EAT ≥ 20) (male in the control group and female in the educated group). Initially, of the total number of students who participated in the research, 19.3% had scores higher than 15 points in the total sum, 58% were female, and 42% were male. These percentages can be compared with what has been found in previous studies, where the risk ranged from 9% to 15% among both male and female university students [32].

It has recently been reported that there has been an increase in body dissatisfaction within the population, particularly among university students in non-Western civilizations [33]. In addition, nutrition students are “fed” during classes with constant messages about proper nutrition. All of these factors can give rise to an obsession with food, proper eating habits, and outward appearance [34]. The importance of research on food habits and potential eating disorders in this population lies in the knowledge of both appropriate and inappropriate strategies and patterns. Students can use all of this information both correctly and incorrectly. The results showed the need to generate longer and more intense programs, since although there was a trend, the intervention time was not sufficient to observe significant changes. For women, the mean score decreased −0.60 in the control group and −0.93 in the educated group. In men, it was −0.59 and −1.11 for the control and educated groups, respectively.

Previous research has shown that nutrition and dietetics students did not have a higher risk of eating disorders and did not differ in the scores of the subscales compared to students from other majors [35]. However, this is not always the case; it has also been found that nutrition and dietetics students had higher levels of eating disorders, especially in terms of dietary restriction [36,37]. Additionally, the authors suggested that students in the field of human nutrition and dietetics may enter these university degrees in an attempt to deal with their own pre-existing struggles with food [36]. Other authors [17] further explored the motivations of students entering dietetics. One of the most important motivations for entering dietetics was a personal experience (including experience with family members or friends) with obesity, EDs, or both, being approximately 30% of participants who entered dietetics for this reason [17].

Other factors attributed to this struggle include daily dealings with food [38], guilt, or self-loathing for deviating from their “healthy food only” diet, fear of consuming unhealthy foods, decreased physical ability to cope with stress, an emotional crisis, or a serious physical or emotional disorder [39]. These studies highlight the unusual relationship that a dietetic professional may have with food, even though it is not explicitly classified as an anxiety disorder according to the Diagnostic and Statistical Manual of Mental Disorders (DSM-V) [40].

Harrer et al. (2019) [10], in their systematic review with a meta-analysis, stated that dissemination of eating disorder interventions in university settings may be a potential way to reduce the incidence of the subthreshold, and potentially comprehensive, eating disorders. For this reason, the present innovative action was developed. Transferring to students the importance of a good dietetic–nutritional education, which is transformed into an adequate nutritional status, will help them to be future promoters of these habits in patients and groups with whom they will work. It has been shown that although dietetic students know that techniques such as vomiting, laxatives, and skipping meals are unhealthy, they use them as a method to lose weight [41]. However, the parameters for optimizing the effects of educational programs have not been studied yet.

Regarding the analysis of body composition, the prevalence of underweight was 13.10%, overweight 13.13%, type I obesity 3.04%, and type II obesity 3.5%. The remaining 78% of students had an adequate weight for their height. It should be noted that the educated group had significantly improved BMI values (*p* < 0.001). Therefore, it can be assumed that young adults in the educated group experienced favorable changes in body composition in terms of weight loss.

A systematic review [42] that analyzed educative interventions related to diet and physical activity in college students showed that out of a total of 12 studies that measured the variable weight and BMI, only four studies offered significant improvements. In all four cases, the population was mostly female. They also highlighted that interventions spanning one college semester or less (≤12 weeks) generally resulted in a greater number of significant outcomes compared to interventions with a duration of more than one semester. This is in line with the study design presented.

As the BMI has an unhealthily low specificity [43], appropriate body composition measurements were performed using the Biodyxpert^®^. From the total, 10.15% of women and 5.58% of men had excessive fat mass (>33% and 20% women and men, respectively). The mean body fat percentage was higher in females than in males, as seen in previous research [44]. After the intervention, women presented a significant improvement in both fat mass in constant hydration (%) and crude fat mass (kg), which did not occur in men. This may be due to what Von Bothmer et al. [45] observed. After conducting a questionnaire examining gender differences in the health habits of university students, they observed that men were less interested in nutrition advice and health-enhancing behaviors, which may affect the impact of the intervention. Interventions should be gender-specific to respond to different needs and interests.

It was observed that the women with the lowest percentage of FM had higher scores on the total punctuation of EAT-26 and dieting scale. This scale is characterized by scrutiny of calorie content, carbohydrates, and sugar content, which is motivated by a desire to be thinner. Therefore, women with a lower percentage of fat mass have greater self-control of food intake and environmental pressure to lose weight. In men, something similar occurs, since the dieting variable and the total sum are significantly and negatively correlated with the BMI variable.

With respect to student nutrition, adherence to DM was adequate in men in both groups. However, women obtained slightly lower scores. It should be noted that in all groups, there were significant improvements. The possible reason for this fact is that in addition to the educational intervention designed, the students attended their corresponding classes, increasing, although in a more indirect and not so specific way, their knowledge of food quality and nutritional habits related to adherence to the Mediterranean diet.

Among men, significant negative correlations were found between the PREDIMED questionnaire and different body composition variables such as weight, BMI, % fat in constant hydration, and kg fat. Therefore, adult young men who are more adherent to the Mediterranean diet have lower body weight and body fat values.

It has been observed that adherence to MD has also been decreasing over the years toward a Western diet model in university students, as indicated by some authors [46,47]. In previous research where they compared nutrition and dietetic students with other health science students, no significant differences have been found in terms of eating habits and healthier lifestyles [48]. Therefore, it seems that although these students have a better knowledge of nutrition, this does not necessarily lead to changes toward a healthier diet and lifestyle.

Therefore, disease prevention requires systematizing and incorporating the contributions of the social sciences such as education. This implies integrating the knowledge on the development of subjectivity accumulated by different scientific disciplines, specially psychology, sociology, and pedagogy [49].

It is necessary to define the mechanisms of the emergence of diseases and the participation that the subjective, the social, and the biological aspects have in them. Moreover, it is essential that the social sciences actively participate in the understanding, definition, and stimulation of disease prevention as much as the medical sciences do. In this way, it is possible to speak of psychosocial disease prevention [49].

The results of this study should be interpreted considering some limitations. First, the study sample was limited; students from other specialties/graduates are required to compare the results. In addition, the results were collected within the population of Alicante. To learn the impact of these programs in other cities, it would be necessary to carry out more studies in different provinces with different eating habits. Future research should consider the analysis of other psychological aspects such as the perception of body image using the body shape questionnaire (BSQ). Possible factors associated with the study variables such as dietary intake and physical exercise were also not considered; therefore, it is recommended that they be included in future research to have a better understanding of the behavior of these variables.

## 5. Conclusions

The educational intervention produced significant improvements in all body composition variables in the women and in the SMM variable in the case of the men; however, it did not produce significant changes in the results of the EAT-26 questionnaire. The results of adherence to the Mediterranean diet improved in both the control and experimental groups. The results obtained were average, with males presenting a higher score, both before and after the intervention. It should be noted that the students with greater control of caloric intake and consumption of carbohydrates and sugars were those with less fat in women, and less weight and BMI in men. In addition, moderate correlations between Mediterranean diet adherence and EAT-26 questionnaire with body composition variables were observed in men. However, in women, the associations observed were small. More social science interventions are needed including measures to assess the impact of nutrition education interventions. In addition, different programs should be made according to the sex of the students, since, as seen in the literature, their interests are not the same.

## Figures and Tables

**Table 1:** Topics in the education program.

Topics in the Education Program	
Nutrition topic	Essential nutrients in the diet of young adults, quantities, and portions. Health consequences of processed products and alcohol consumption. Healthy snacking.
Healthy lifestyle (physical activity)	Influence of physical activity on physical and intellectual development and healthy well-being. Recommendations based on the WHO guidelines [28].
Risk behaviors and eating disorders	Factors that influence body perception. What promotes and reduces positive body image and how can we enforce health-promoting factors? Strengthen acceptance and love for individual differences, defining characteristics of self and friends.

**Table 2:** Descriptive statistics (mean ± standard deviation) of the body composition of the women.

	CONTROL GROUP				EDUCATED GROUP				ANOVA					
	PRE		POST		PRE		POST		Effect Time			Effect Time × Group		
	Mean	SD	Mean	SD	Mean	SD	Mean	SD	F	*p*	η^2^p	F	*p*	η^2^p
Weight	58.7	10.4	57.9	12.5	59.9	11.1	59.0	11.7	10.00	0.002	0.065	6.36	0.013	0.042
BMI	21.9	3.24	21.6	4.13	22.9	4.17	22.5	4.30	10.62	0.001	0.069	7.23	0.008	0.048
FM constant hydration (%)	26.4	5.62	26.4	5.94	27.6	6.13	26.7	6.61	9.83	0.002	0.063	6.65	0.011	0.044
Crude FM (Kg)	16.4	6.74	16.4	6.99	28.2	6.47	27.3	6.95	10.10	0.002	0.065	9.20	0.003	0.060
SMM (Kg)	21.3	3.06	21.3	2.99	12.5	1.77	14.2	4.77	11.3	0.001	0.072	10.2	0.002	0.066
ASMM (Kg)	16.2	2.33	16.2	2.27	21.4	2.75	24.5	8.11	11.1	0.001	0.071	10.6	0.001	0.068
Hydration without grass (%)	68.5	2.12	68.6	2.53	68.9	2.29	67.7	12.5	10.02	0.002	0.065	6.82	0.010	0.045
Total Water (L)	29.6	3.49	29.6	3.39	31.8	5.30	32.2	5.42	8.58	0.004	0.057	6.71	0.011	0.045

SD = standard deviation; n = number of subjects per group; FM = fat mass; % = percentage; kg = kilograms; SMM = skeletal muscle mass; ASMM = appendicular skeletal muscle mass; L = liters; F = variance quotient; η^2^p = partial eta squared.

**Table 3:** Descriptive statistics (mean ± standard deviation) of the body composition of the men.

	CONTROL GROUP				EDUCATED GROUP				ANOVA					
	PRE		POST		PRE		POST		Effect Time			Effect Time × Group		
	Mean	SD	Mean	SD	Mean	SD	Mean	SD	F	*p*	η^2^p	F	*p*	η^2^p
Weight	68.8	12.2	68.7	12.3	72.6	10.5	71.6	11.1	3.76	0.058	0.071	2.73	0.105	0.053
BMI	22.3	3.34	22.3	3.45	24.2	2.85	23.9	3.09	3.54	0.066	0.069	2.90	0.095	0.057
FM constant hydration (%)	16.4	7.56	16.3	8.05	17.7	6.31	16.7	6.65	3.63	0.063	0.070	2.60	0.114	0.051
Crude FM (Kg)	11.8	7.19	11.8	7.65	18.0	6.57	16.9	6.87	4.00	0.051	0.077	3.22	0.079	0.063
SMM (Kg)	29.8	4.29	29.9	4.55	16.8	1.47	18.8	4.83	4.19	0.046	0.080	3.85	0.055	0.074
ASMM (Kg)	22.8	3.33	22.9	3.51	32.0	3.23	35.5	8.46	3.61	0.064	0.070	3.49	0.068	0.068
Hydration without grass (%)	70.3	1.34	70.5	2.29	69.8	1.86	65.5	19.4	4.09	0.049	0.082	3.01	0.090	0.061
Total Water (L)	40.1	5.29	40.2	5.49	37.1	6.67	37.7	6.60	3.75	0.059	0.075	2.24	0.141	0.046

SD = standard deviation; n = number of subjects per group; FM = fat mass; % = percentage; kg = kilograms; SMM = skeletal muscle mass; ASMM = appendicular skeletal muscle mass; L = liters; F = variance quotient; η^2^p = partial eta squared.

**Table 4:** Descriptive values (mean and standard deviation) of the PREDIMED questionnaire by group and sex.

CONTROL GROUP				EDUCATED GROUP					ANOVA				
PRE		POST		PRE		POST		Effect Time			Effect Time × Group		
Mean	SD	Mean	SD	Mean	SD	Mean	SD	F	*p*	η^2^p	F	*p*	η^2^p
FEMALES													
6.67	2.82	7.67	2.82	6.78	2.83	8.09	2.97	400.87	< 0.001	0.774	7.13	0.009	0.057
MALES													
7.26	3.19	8.26	3.19	7.15	2.62	8.23	2.61	135.39	0.001	0.069	0.18	0.008	0.048

SD = standard deviation; F = variance quotient; η^2^p = partial eta squared.

**Table 5:** Correlations between variables in women.

	PMED	Dieting	B-F p	O-C	EAT 26
Weight (kg)	0.040	−0.177	0.034	−0.202 *	−0.168
BMI (kg/m^2^)	0.087	−0.179	0.023	−0.159	−0.157
FMCH (%)	0.017	−0.200 *	−0.010	−0.165	−0.181 *
CFM (Kg)	0.069	−0.095	−0.064	−0.137	−0.124
SMM (Kg)	−0.011	−0.076	0.120	−0.163	−0.069
ASMM (Kg)	0.117	0.069	−0.025	−0.110	−0.006
HWF (%)	0.010	0.128	0.010	0.086	0.109
TW (L)	−0.021	0.032	0.015	−0.096	−0.011

* *p* < 0.05; BMI = body mass index; FMCH = fat mass at constant hydration; CFM = crude fat mass; SMM = skeletal muscle mass; ASMM = appendicular skeletal muscle mass; HWF = hydration without fat; TW = total water; PMED = PREDIMED; B-F p = bulimia and food preoccupation; O-C = oral control.

**Table 6:** Correlations between the variables in men.

	PMED	Dieting	B-F p	O-C	EAT 26
Weight (kg)	−0.463 *	−0.314 *	−0.001	−0.133	−0.231
BMI (kg/m^2^)	−0.475 **	−0.409 *	−0.104	−0.269	−0.375 *
FMCH (%)	−0.359 *	−0.226	−0.031	−0.147	−0.196
CFM (Kg)	−0.421 *	−0.279	−0.095	−0.095	−0.234
SMM (Kg)	0.023	0.191	0.309	−0.165	0.175
ASMM (Kg)	0.080	0.071	−0.077	0.029	0.021
HWF (%)	0.113	0.164	0.144	0.210	0.227
TW (L)	−0.211	−0.195	0.164	−0.239	−0.133

* *p* < 0.05, ** *p* < 0.001; BMI = body mass index; FMCH = fat mass at constant hydration; CFM = crude fat mass; SMM = skeletal muscle mass; ASMM = appendicular skeletal muscle mass; HWF = hydration without fat; TW = total water; PMED = PREDIMED B-F p = bulimia and food preoccupation; O-C = oral control.

## Data Availability

Not applicable.
